# A Novel Multimodal Implementation of a Foundation Artificial Intelligence Model Using Optic Nerve Head Fundus Photographs and OCT Imaging for Glaucoma Detection

**DOI:** 10.1016/j.xops.2025.101012

**Published:** 2025-11-17

**Authors:** Benton Chuter, Vedant Joshi, Shahin Hallaj, Evan Walker, Christopher Bowd, Akram Belghith, Michael H. Goldbaum, Andrzej Grzybowski, Massimo A. Fazio, Christopher A. Girkin, C. Gustavo De Moraes, Jeffrey M. Liebmann, Robert N. Weinreb, Linda M. Zangwill, Mark Christopher

**Affiliations:** 1 Division of Ophthalmology Informatics and Data Science, Viterbi Family Department of Ophthalmology, Shiley Eye Institute, University of California, San Diego, La Jolla, California; 2 Hamilton Glaucoma Center, Viterbi Family Department of Ophthalmology, Shiley Eye Institute, University of California, San Diego, La Jolla, California; 3 Department of Ophthalmology and Vision Sciences, University of Alabama at Birmingham, Birmingham, Alabama; 4 Department of Ophthalmology, Harkness Eye Institute, Bernard and Shirlee Brown Glaucoma Research Laboratory, New York, New York; 5 Department of Ophthalmology, Uniwersytet Warminsko-Mazurski w Olsztynie, Olsztyn, Poland; 6 Institute for Research in Ophthalmology, Foundation for Ophthalmology Development, Poznan, Poland

**Keywords:** Artificial intelligence, Foundation model, Fundus photographs, Multimodal, OCT

## Abstract

**Purpose:**

To compare the performance of unimodal and multimodal implementation of the self-supervised learning model RETFound in detecting glaucoma using color fundus photographs (CFPs) and OCT images, and to assess its generalizability across different ethnicities, age groups, and disease severities.

**Design:**

Evaluation of a diagnostic technology.

**Subjects, Participants, and Controls:**

Fourteen thousand five hundred ten CFPs and 32 640 OCTs from 1948 eyes of 1098 participants (60.8% glaucoma, 39.2% healthy) from the Diagnostic Innovations in Glaucoma Study and the African Descent and Glaucoma Evaluation Study were included. Glaucoma was defined as photograph-based glaucomatous optic neuropathy with or without repeatable glaucoma visual field damage.

**Methods:**

A multimodal RETFound model was developed using paired CFPs and OCT images. The model was compared to unimodal RETFound models using solely CFP or OCT images. Performance was also stratified by race (Black vs. White), age (<60 vs. ≥60 years), and disease severity (mild vs. moderate-to-severe glaucoma).

**Main Outcome Measures:**

Diagnostic accuracy of unimodal and multimodal RETFound models using CFP and OCT for detecting glaucoma was assessed using the area under the receiver operating characteristic curve (AUC), precision, and recall.

**Results:**

The multimodal model for glaucoma detection achieved an AUC of 0.94 (95% confidence interval: 0.91–0.97), significantly outperforming the CFP unimodal model (AUC 0.86 [95% confidence interval: 0.81–0.89], *P* < 0.001) but not the OCT unimodal model (AUC 0.93 [95% confidence interval: 0.90–0.96], *P* = 0.47). Precision and recall were higher (0.96 and 0.87, respectively) for the multimodal model compared with the CFP model (0.92 and 0.69) across all subgroups. No significant differences based on race or age were found in either unimodal or multimodal glaucoma detection models. All models exhibited better performance in detecting moderate-to-severe glaucoma than mild glaucoma, with significant differences in the unimodal CFP (*P* = 0.002) and OCT (*P* = 0.005) models.

**Conclusions:**

The multimodal RETFound model demonstrated improved diagnostic ability compared with the CFP unimodal model but did not significantly outperform the OCT unimodal model in glaucoma detection. As clinical implementation of a unimodal artificial intelligence (AI) model is easier than a multimodal counterpart, our results suggest unimodal OCT AI models may be sufficient for detecting glaucoma.

**Financial Disclosure(s):**

Proprietary or commercial disclosure may be found in the Footnotes and Disclosures at the end of this article.

Glaucoma is a leading cause of vision loss associated with progressive optic neuropathy; accurate diagnosis and monitoring is required to prevent permanent blindness.^1^, ^2^, ^3^ Both color fundus photographs (CFPs) and OCT images play a crucial role in the clinical management of glaucoma, enabling early detection and regular surveillance.^4^, ^5^, ^6^ However, dependence on specialist clinicians to analyze CFPs and OCT images presents significant demands on clinician time as well as challenges with consistency in evaluations among different physicians.^7^

In recent years, artificial intelligence (AI) systems have been explored to potentially address these issues by serving as automated clinical decision support systems to assist in interpreting CFPs and OCT images for the monitoring and diagnosis of glaucoma.^1^^,^^8^^,^^9^ However, training these models typically depends upon extensive high-quality labeled data sets, which may be prohibitively resource-intensive to produce.^9^, ^10^, ^11^, ^12^ As such, this dependence on expert-annotated data may restrict scalability and limit viability in varied clinical environments.^10^^,^^11^

Self-supervised learning (SSL) is a relatively recent machine learning approach that can overcome this label barrier.^13^, ^14^, ^15^, ^16^, ^17^ By extracting features without predefined ground truth labels, SSL generates flexible feature representations for multiple applications. Leveraging extensive collections of unlabeled data, this method can train generalizable models that can be adapted to various tasks and often surpass the performance of task-specific supervised learning approaches.^18^^,^^19^ This characteristic makes SSL-based models a promising option for medical applications.^16^^,^^20^^,^^21^

RETFound is an SSL-based foundation model that applies this approach to 2 ophthalmic imaging modalities: CFP and OCT.^22^ RETFound was trained on roughly 1.7 million ophthalmic images and serves as a foundational model that can be adapted or fine-tuned for specific tasks without requiring prohibitively large training data sets.^22^ Preliminary evaluations demonstrated RETFound's utility across various diseases, tasks, and imaging modalities.^22^ Some literature, including the original RETFound paper, has demonstrated the advantages of using either CFP or OCT imaging as the sole model input.^22^^,^^23^ Zhang et al^24^ found that the unimodal CFP version of RETFound significantly improved the sensitivity and specificity of community-based eye disease screenings for age-related macular degeneration, pathological myopia, and diabetic retinopathy compared with commercial models and demonstrated better generalization in both urban and rural settings in China. More recently, we demonstrated RETFound’s ability to achieve high performance in glaucoma detection when using relatively few CFPs to finetune the model.^25^ Another recent study also showed high RETFound performance in single-output tasks involving feature extraction from CFPs.^26^

However, independent evaluation of RETFound remains limited. Furthermore, RETFound’s performance has yet to be characterized when used in a multimodal capacity involving both CFPs and OCT imaging for glaucoma detection, an approach which may further improve performance. Using CFPs and OCT together potentially provides complementary information to the models. Color fundus photographs provide a full color view to help characterize the optic nerve head (ONH) appearance, while OCT imaging provides a 3-dimensional view of retinal layers and structure. Combining these modalities may provide a more complete representation of the relevant features and enable more accurate glaucoma classification. The goal of this work is to assess the performance of a multimodal RETFound approach to detect glaucoma using both CFP and OCT imaging. Generalizability across patient demographics and disease severity is also evaluated.

## Methods

### CFP and OCT Datasets

Optic nerve head CFPs and OCTs from the Diagnostic Innovations in Glaucoma Study (DIGS) (clinicaltrials.gov identifier: NCT00221897)^27^ and the African Descent and Glaucoma Evaluation Study (ADAGES) (clinicaltrials.gov identifier: NCT00221923)^28^ comprised the study’s image set. The DIGS and ADAGES studies are joint ventures that include a broad range of participants of African, European, and Asian descent from the University of California, San Diego Hamilton Glaucoma Center and Viterbi Family Department of Ophthalmology, the University of Alabama at Birmingham Department of Ophthalmology, and the Edward S. Harkness Eye Institute at Columbia University Medical Center. These studies’ patient enrollment and methods were approved by the institutional review boards of each participating institution, adhering to the Declaration of Helsinki and the Health Insurance Portability and Accountability Act. All participants provided written informed consent at the time of recruitment. Although the methods have been extensively documented in prior publications, pertinent details are summarized here.^28^

All participants underwent regular semiannual OCT imaging and visual field (VF) testing as well as annual optic disc stereophotograph acquisition. Visual field tests were performed using the Humphrey Field Analyzer II with a 24-2 Swedish Interactive Thresholding Algorithm standard test pattern, excluding tests with >33% in fixation losses, false negatives, or false positives. Glaucoma VF damage was defined as ≥3 consecutive glaucomatous 24-2 VF results (pattern standard deviation ≥95th percentile or glaucoma hemifield test outside normal limits).^28^ Color fundus photographs were classified as glaucomatous versus healthy by masked review by 2 experienced graders. Disagreements were resolved by adjudication or consensus.

At baseline, study requirements for healthy participants in DIGS and ADAGES were as follows: 20/40 or better best-corrected visual acuity, CFPs without evidence of glaucomatous damage, reliable standard automated perimetry VF tests without evidence of glaucomatous VF damage (glaucoma VF damage), and intraocular pressure (IOP) of <22 mmHg.^28^ Glaucomatous eyes were defined as those with glaucomatous optic neuropathy based on review of CFPs. Color fundus photograph review was performed by 2 expert reviewers, with a senior reviewer adjudicating disagreements. This CFP-based glaucomatous optic neuropathy status alone was used to classify eyes as glaucomatous versus nonglaucomatous. The VF mean deviation (MD) in tests performed nearest to the time of image capture and within 1 year was used to estimate the severity of glaucomatous functional damage at the time of imaging.

Optic nerve head radical circle scans were obtained on all participants at each biannual visit, consisting of 24 high-resolution ONH radial scans and 3 circle scans, using the Spectralis Glaucoma Module Premier Edition (version 6.10; Heidelberg Engineering Inc). At the University of California, San Diego Imaging Data Evaluation and Analysis Reading Center, OCT images underwent quality assessment, and low-quality images were excluded from further analyses. The exclusion criteria included quality score <15 or poor centering of the image, poor focus, artifacts, and segmentation errors that could not be manually corrected. All 3 retinal nerve fiber layer circle b-scans from a single ONH radical circle image were included in the model without segmentation.

The OCT data set included 32 640 OCT images from 1163 glaucoma eyes of 694 participants and from 766 healthy eyes from 429 participants. The CFP data set contained 14 510 stereo CFPs sourced from 1948 eyes of 1098 participants (Table S1, available at www.ophthalmologyscience.org).

### Multimodal Data Set Construction

**Figure 1:**
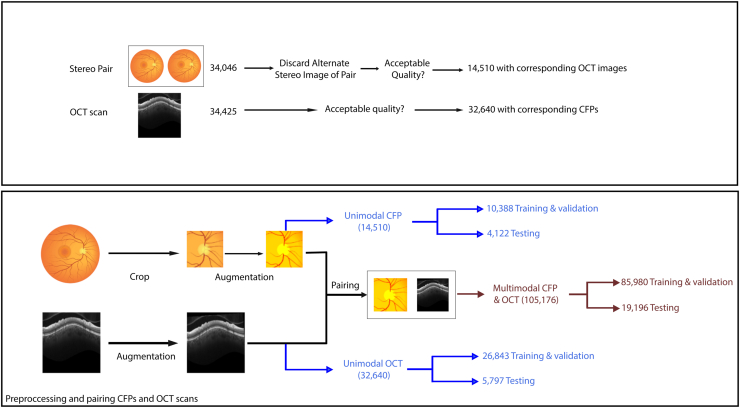
Schematic illustrating dataset construction and splitting into training/testing cohorts. CFP = color fundus photograph.

The DIGS/ADAGES study acquired stereophotographs annually and OCT images semiannually. One CFP of each stereopair was randomly selected and then automatically cropped to a standard format (Fig 1), as previously described.^29^ The CFP cropped region covered an area centered on the ONH with a width and height of 2.5 times the optic disc diameter. Extracting this region helps provide a standardized view of the ONH regardless of the specific camera or imaging set-up and we have previously achieved high deep learning glaucoma detection accuracy using this approach.^30^^,^^31^ After the standardized cropping, a reviewer manually inspected a sample of the CFPs to ensure accurate cropping. Both CFPs and OCT images were resized to a uniform 224 × 224 pixels, the expected input size of RETFound.

**Figure 2:**
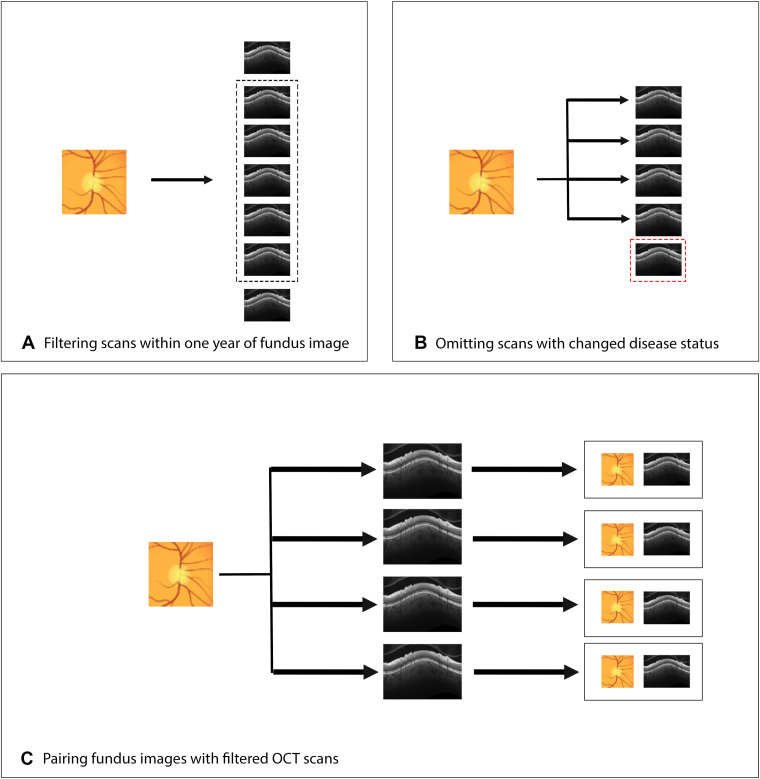
Schematic illustrating the pairing between color fundus photographs and OCT imaging. (A) All OCT captured within 1 year of a color fundus photograph were identified. (B) OCT captured after any change in disease status (e.g., conversion to glaucoma) were excluded. (C) Color fundus photograph – OCT pairs were constructed from the remaining images.

For each CFP for a given eye, all corresponding OCT images taken within 1 year were matched to generate CFP-OCT pairs (Fig 2). Because a single CFP could be paired with multiple OCT images acquired during this period, the total number of multimodal training images may exceed the counts used in CFP-only or OCT-only models. The disease status (healthy or glaucoma) of the eye at the time of the CFP was used for characterizing disease status of all associated pairs. OCT images for which disease status did not match that of the CFP status, due to changes within the 1-year time frame, were not paired to that CFP. Otherwise, this implementation assumes that the disease status of a patient’s eye does not meaningfully progress within 1 year to sufficiently decouple the CFP and OCT images. One benefit of this approach is model exposure to “real-world augmentation,” that is, CFP and OCT images appear in all possible pairings that meet the time window cutoff, rather than in just a single CFP-OCT pair. This provides the multimodal model with a greater sample of potential pairs to learn from, potentially enhancing generalizability. The resulting data set included 1098 unique patients and was split into train/validation (n = 898) and test (n = 200) subsets. The data set included 85 980 CFP-OCT pairs (44 716 right eye; 41 264 left eye) for training/validation and 19 196 pairs (10 365 right eye; 8831 left eye) for testing (Fig 1). Altogether, these multimodal pairs represent all possible matches between the 2 modalities within the constraint of 1 year and same disease label (glaucomatous vs. healthy) set.

Image augmentation was then implemented to increase diversity of the training and validation data sets, enabling the model to better handle variations in new, unseen images by simulating clinically relevant distortions. For CFPs, random augmentations included horizontal flipping, horizontal and vertical translation, rotation, crop, rescaling, and pixel intensity noise. For OCT, random small rotations, vertical translations, and pixel intensity noise were applied. Small random horizontal translations with a roll around effect (i.e., A-scans shifted off one side of the circle scan are moved to the other side to mimic small rotations of the circle scans) were also applied. For each OCT image and CFP pair used in the multimodal model, horizontal flipping augmentations were performed in concert to help maintain positional relationships in the CFP and OCT images.

### Multimodal RETFound

RETFound uses a SSL approach to train ophthalmic foundation models on a database of approximately 1.7 million images.^13^^,^^18^^,^^19^^,^^22^ Our multimodal approach builds upon the original RETFound model by proposing a single model that uses both OCT and CFP image simultaneously to detect glaucoma. For these analyses, we trained and evaluated 3 models: (1) unimodal RETFound CFP model, (2) unimodal RETFound OCT model, and (3) multimodal CFP + OCT RETFound model. For the unimodal cases, a final fully connected layer and a binary classification output layer were added to the published RETFound models. Additional fine-tuning was performed on the corresponding training data sets (CFP and OCT). For the multimodal case, a combined model was constructed by concatenating outputs from each unimodal model and adding additional fully connected and classification layers. This modal was then fine-tuned on the combined CFP-OCT training data set.

For both cases, some common training steps were applied. For each model, training was stopped after no improvement for 10 continuous epochs. During training, area under the curve receiver operating characteristic curve (AUC) were computed on the validation set and the model with the highest AUC on the validation split was selected for evaluation test set. Because the data set was somewhat imbalanced (more glaucoma vs. healthy cases), focal loss was used to weight training errors and examples based on class frequency.

During fine-tuning of the unimodal models, an additional fully connected sigmoid layer consisting of 1024 nodes was added to the RETFound model to produce a final output. Both the original RETFound and added sigmoid layer weights were allowed to update during training. For the multimodal case, outputs from the final unimodal sigmoid layers were concatenated and passed through another rectified linear units and fully connected sigmoid layer to produce a final classification. Here again, all weights in the model were allowed to update during training. Training was done using stochastic-gradient descent with momentum (0.9), label smoothing (0.1), batch size 64, a cosine learning-rate schedule that rose to 5 × 10^-4^ over the first 5 epochs before decaying to 1 × 10^-6^ by epoch 100, L2 weight-decay (1 × 10^-5^), and automatic mixed-precision to accelerate training. Additional details can also be found in Table S1, available at www.ophthalmologyscience.org.

### Model Evaluation

Area under the receiver operating characteristic curve as well as precision and recall at 0.80, 0.90, and 0.95 specificity thresholds were calculated to evaluate model performance. Model generalizability was evaluated by stratifying the results based on race (Black/African American vs. White), age (<60 years vs. ≥60 years), and severity of glaucoma (VF MD > -6.0 decibels vs. MD ≤ -6.0 decibels). Model performance was also stratified based on use of all image pairs within the year time frame, or only the most recent OCT image corresponding to a specific CFP.

To provide further insight into model predictions, a visualization technique was used to generate heatmaps that identify key regions that contributed to model decisions.^32^ This identifies image regions that have a large impact on model predictions and has demonstrated superior performance compared with other popular visualization techniques.^33^ A random subset of CFP-OCT pairs that included true-positive, true-negative, false-positive, and false-negative predictions by the multimodal model was evaluated in this way to generate a selection of examples for review.

## Results

This analysis included 14 510 CFPs and 32 640 OCT images from 1098 subjects and 1948 eyes, divided into subsets for testing (200 subjects and 375 eyes) and training/validation (898 subjects and 1573 eyes), as shown in Table 2. The average baseline age of participants in the study is 60.3 years. Females (n = 646, 58.8%) outnumber males (n = 452, 41.2%). The majority of the study population is White (n = 597, 54.4%), followed by Black/African American (n = 427, 38.9%), Asian (n = 57, 5.2%), and other/not disclosed (n = 25, 2.3%) individuals. Other characteristics such as VF MD, axial length, spherical equivalent, IOP, and central corneal thickness are documented for the training, validation, and test sets in Table 2.

**Table 2:** Summary of the Training/Validation and Testing Data Sets Used for This Study

Measurement	Train/Validation		Test	
	Glaucoma	Healthy	Glaucoma	Healthy
Sample sizes				
Patients (eyes) (n)	557 (955)	341 (618)	111 (205)	89 (170)
Fundus photographs (n)	7704	2684	2865	1257
OCT images (n)	20 432	6411	4130	1667
Patient-level characteristics				
Baseline age (years)	66.7 (65.7, 67.6)	51.6 (50.0, 53.2)	62.9 (60.4, 65.4)	50.0 (47.0, 53.0)
Last age (years)	70.6 (69.6, 71.5)	53.2 (51.5, 54.9)	71.9 (69.6, 74.3)	56.4 (53.0, 59.7)
Race (%)				
Asian	36 (6.5%)	13 (3.8%)	4 (3.6%)	4 (4.5%)
Black or African American	211 (37.9%)	139 (40.8%)	45 (40.5%)	32 (36.0%)
White	302 (54.2%)	181 (53.1%)	62 (55.9%)	52 (58.4%)
American Indian, Hawaiian, Pacific	8 (1.6%)	8 (2.3%)	4 (3.6%)	5 (5.6%)
Islander, unknown, or not reported				
Sex (%)				
Female	303 (54.4%)	211 (61.9%)	68 (61.3%)	64 (71.9%)
Male	254 (45.6%)	130 (38.1%)	43 (38.7%)	25 (28.1%)
Ethnicity (%)				
Hispanic	9 (1.6%)	12 (3.5%)	3 (2.7%)	4 (4.5%)
Not Hispanic	508 (91.2%)	285 (83.6%)	103 (92.8%)	70 (78.7%)
Unknown or declined to answer	40 (7.2%)	44 (12.9%)	5 (4.5%)	15 (16.9%)
Baseline visit eye-level characteristics				
24-2 VF MD (dB)	–5.06 (–6.90, –3.21)	–0.38 (–4.87, 4.10)	–4.55 (–5.43, –3.67)	–0.83 (–2.96, 1.29)
Axial length (mm)	24.2 (24.1, 24.3)	23.9 (23.8, 24.0)	24.1 (23.9, 24.3)	23.9 (23.6, 24.1)
Spherical equivalent (D)	–3.56 (–3.96, –3.17)	–2.38 (–2.82, –1.94)	–0.57 (–0.91, –0.23)	–0.55 (–0.92, –0.18)
IOP (mmHg)	15.36 (15.03, 15.69)	14.73 (14.31, 15.15)	17.98 (17.14, 18.82)	16.08 (15.16, 17.01)
CCT (μm)	538.2 (534.9, 541.5)	542.8 (539.0, 546.6)	544.1 (537.8, 550.4)	547.1 (540.6, 553.6)

CCT = central corneal thickness; D = diopters; dB = decibels; IOP = intraocular pressure; MD = mean deviation; VF = visual field.

Table 3 reports the performance (AUC, precision, recall) of the unimodal and multimodal models. For the overall cohort, the single-modality CFP model achieved an AUC (95% confidence interval) of 0.86 (0.81, 0.89), a precision of 0.92 (0.88, 0.95), and a recall of 0.69 (0.63, 0.74). The single-modality OCT model had higher performance with an AUC of 0.93 (0.90, 0.96), a precision of 0.96 (0.93, 0.98), and a recall of 0.81 (0.75, 0.86). The multimodal CFP + OCT model had the highest performance with an AUC of 0.94 (0.91, 0.97), a precision of 0.96 (0.92, 0.98), and a recall of 0.87 (0.82, 0.91). The multimodal AUC was significantly (*P* < 0.001) better than the unimodal CFP model, but not significantly better than unimodal OCT model (*P* = 0.47).

**Table 3:** Performance of the Unimodal and Multimodal Models on the Testing Data Set

Modality	AUC (95% CI)	80% Specificity		90% Specificity		95% Specificity		*P* Value∗
		Precision (95% CI)	Recall (95% CI)	Precision (95% CI)	Recall (95% CI)	Precision (95% CI)	Recall (95% CI)	
CFP	0.86 (0.81, 0.89)	0.56 (0.47, 0.65)	0.56 (0.47, 0.65)	0.67 (0.57, 0.74)	0.59 (0.47, 0.69)	0.76 (0.67, 0.82)	0.47 (0.37, 0.57)	<0.001
OCT	0.93 (0.90, 0.96)	0.61 (0.51, 0.69)	0.61 (0.51, 0.69)	0.73 (0.63, 0.79)	0.78 (0.66, 0.87)	0.81 (0.74, 0.86)	0.64 (0.51, 0.76)	0.47
Multimodal	0.94 (0.91, 0.97)	0.58 (0.47, 0.67)	0.58 (0.47, 0.67)	0.71 (0.61, 0.79)	0.82 (0.70, 0.91)	0.81 (0.72, 0.86)	0.70 (0.57, 0.83)	-

AUC = area under the receiver operating characteristic curve; CFP = color fundus photograph; CI = confidence interval.

∗ Comparison to multimodal model.

**Figure 3:**
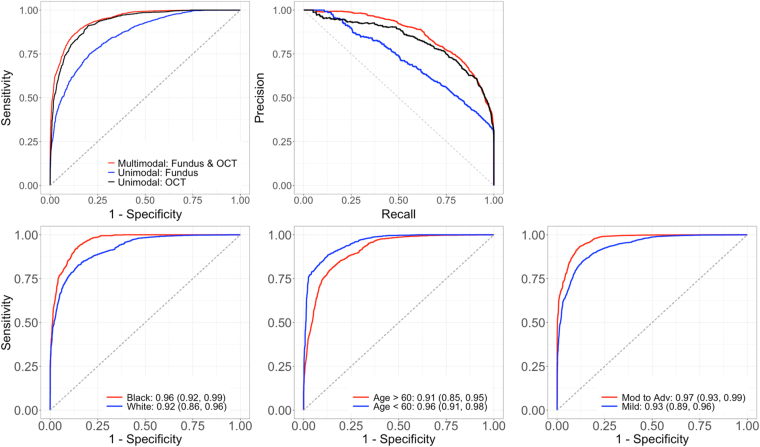
(Top row) Receiver operating characteristic and precision-recall curves for the unimodal and multimodal models. (Bottom row) Receiver operating characteristic curves for the multimodal model stratified by race, age, and disease severity.

To assess the generalizability of the models, the impact of race, age, and disease severity were also evaluated using stratified analyses (Fig 3, Table 4). With respect to race, only Black/African American and White cohorts were considered because other groups had too few participants. In both Black and White populations, the multimodal model attained the highest AUCs of 0.96 and 0.92, respectively, significantly surpassing the CFP model (*P* = 0.01 for Black/African American; *P* = 0.02 for White), but not the OCT model (*P* = 0.39 for Black/African American; *P* = 0.96 for White). Differences in model performance between racial groups were not statistically significant (all *P* values >0.16). Similarly, in age-stratified groups (<60 and ≥60 years), the multimodal model outperformed (AUC = 0.91 for >60, 0.96 for <60) single-modality models, with significant differences over the CFP model (*P* = 0.007 for >60 and *P* = 0.002 for <60), but not the OCT model (*P* = 0.56 for >60 and *P* = 0.20 for <60). The differences across age groups were not significant. When stratified by disease severity, the multimodal model outperformed (AUC = 0.93 for mild, 0.97 for moderate-to-severe), significantly surpassing the CFP model in mild cases (*P* = 0.001 for mild; *P* = 0.07 for moderate-to-severe), but not the OCT model (*P* = 0.42 for mild; *P* = 0.96 for moderate-to-severe). Both unimodal models performed significantly better in moderate-to-severe cases, but there was no significant difference across disease severity for the multimodal model.

**Table 4:** Performance of Unimodal and Multimodal Models Stratified by Patient Race, Age, and Disease Severity for n Subjects

	Modality	Black (n = 76)				White (n = 113)				Intergroup *P* Values
		AUC	*P* Value∗	95% Specificity		AUC	*P* Value∗	95% Specificity		Black vs. White
				Precision	Recall			Precision	Recall	
Race	CFP	0.88 (0.83, 0.92)	0.010	0.95 (0.87, 0.99)	0.50 (0.40, 0.59)	0.83 (0.76, 0.88)	0.019	0.97 (0.94, 0.98)	0.41 (0.33, 0.48)	0.158
	OCT	0.94 (0.89, 0.97)	0.376	0.97 (0.93, 0.99)	0.77 (0.66, 0.86)	0.92 (0.86, 0.96)	0.948	0.98 (0.95, 0.99)	0.64 (0.55, 0.73)	0.496
	Multimodal	0.96 (0.92, 0.99)	-	0.98 (0.94, 0.99)	0.83 (0.71, 0.92)	0.92 (0.86, 0.96)	-	0.98 (0.94, 0.99)	0.61 (0.52, 0.71)	0.176

	Modality	>60 years (n = 131)				<60 years (n = 77)				>60 vs. <60
		AUC	*P* value∗	95% specificity		AUC	*P* value∗	95% specificity		
				Precision	Recall			Precision	Recall	
Age	CFP	0.82 (0.76, 0.87)	0.007	0.98 (0.95, 0.99)	0.41 (0.35, 0.47)	0.84 (0.77, 0.90)	0.002	0.91 (0.81, 0.96)	0.38 (0.26, 0.50)	0.529
	OCT	0.89 (0.83, 0.93)	0.508	0.99 (0.97, 1.00)	0.57 (0.49, 0.64)	0.91 (0.84, 0.96)	0.206	0.91 (0.78, 0.98)	0.65 (0.47, 0.79)	0.550
	Multimodal	0.91 (0.85, 0.95)	-	0.99 (0.97, 1.00)	0.66 (0.57, 0.75)	0.96 (0.91, 0.98)	-	0.94 (0.83, 0.99)	0.74 (0.63, 0.86)	0.183

	Modality	Mild glaucoma (n = 94)				Moderate to severe (n = 56)				Mild vs. moderate to severe *P* value
		AUC	*P* value∗	95% specificity		AUC	*P* value∗	95% specificity		
				Precision	Recall			Precision	Recall	
Disease severity	CFP	0.84 (0.79, 0.88)	<0.001	0.94 (0.90, 0.97)	0.36 (0.30, 0.43)	0.93 (0.88, 0.95)	0.128	0.89 (0.81, 0.95)	0.68 (0.59, 0.77)	0.002
	OCT	0.91 (0.87, 0.94)	0.429	0.96 (0.93, 0.98)	0.62 (0.54, 0.69)	0.97 (0.95, 0.99)	0.961	0.94 (0.88, 0.97)	0.86 (0.78, 0.92)	0.005
	Multimodal	0.93 (0.89, 0.96)	-	0.97 (0.93, 0.99)	0.66 (0.58, 0.74)	0.97 (0.93, 0.99)	-	0.94 (0.86, 0.98)	0.85 (0.70, 0.94)	0.102

AUC = area under the receiver operating characteristic curve; CFP = color fundus photograph.

∗ Denotes *P* value when comparing modality of that row (CFP only or OCT only) to multimodal version.

**Figure 4:**
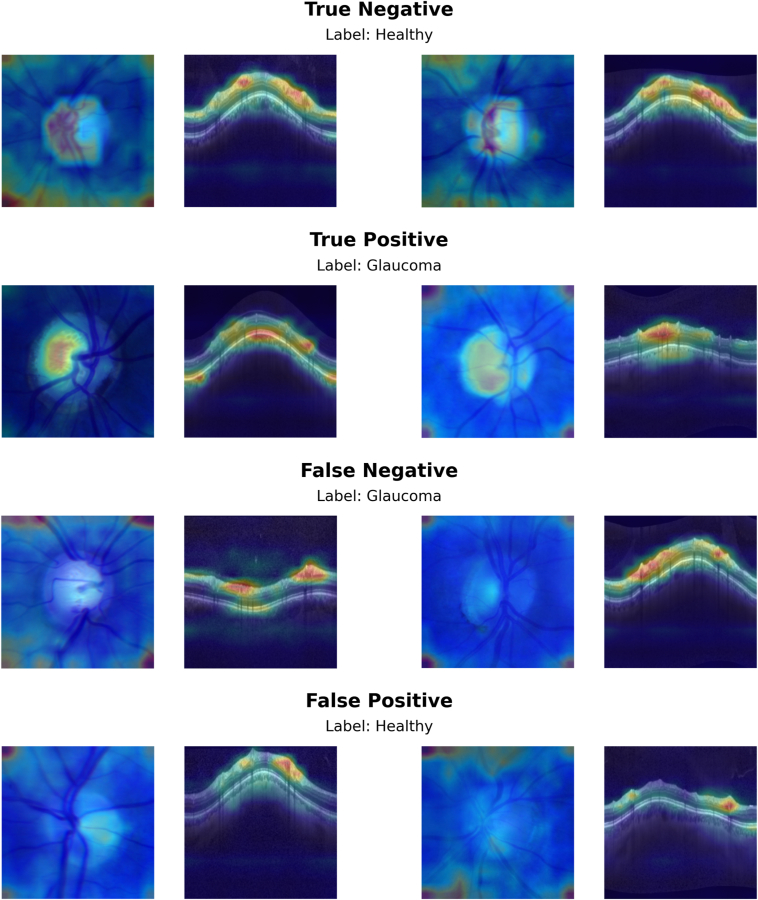
Heatmaps indicating areas of the input images that had a large impact on model predictions.

Heatmaps were generated for a random sample of CFP-OCT pairs (Fig 4) to help identify informative image regions. With respect to OCT inputs, the model seemed to be primarily focused corresponding to the retinal nerve fiber layer and, to a lesser extent, areas near the retinal pigmented epithelium and choroid. For CFPs, there seemed to be more variability in the areas focused on by the model. Heatmaps for correctly predicted CFP input highlighted regions within the ONH, while heatmaps for incorrect predictions focused more on peripheral regions near image corners, potentially relying on artifacts visible there.

## Discussion

We found that the multimodal RETFound model described here did achieve significantly higher performance compared with the unimodal CFP model for glaucoma detection (AUC of 0.94 vs. 0.86, *P* < 0.001). However, it did not significantly outperform the unimodal OCT approach (AUC of 0.93, *P* = 0.47). This is one of the first published studies to evaluate RETFound performance in a multimodal approach using both CFP and OCT imaging to identify glaucoma. We hypothesized that combining these modalities in a unified multimodal RETFound model may help AI models compensate for weaknesses of each individual modality and improve detection. However, our results suggest that the diagnostic accuracy performance of RETFound unimodal model using OCT alone is not significantly lower than the multimodal RETFound model using both CFP and OCT. As implementation of a unimodal AI model is easier than a multimodal counterpart, our results have clear implications for translation of AI glaucoma models into clinical practice; unimodal OCT AI models may be sufficient.

These findings were similar in the analyses stratified by race, age, and disease severity. The multimodal model consistently had significantly higher performance than the unimodal CFP model and model but not significantly higher performance than the unimodal OCT. The stratified analysis also revealed consistent results across the patient groups within each model. All 3 approaches had similar performance in Black/African American compared with the White subgroup as well as older (>60 years) compared with the younger (<60 years) cohort; differences did not reach statistical significance. With respect to disease severity, all models had higher accuracy in identifying moderate-to-severe compared with mild glaucoma, significantly better in the unimodal CFP and OCT cases.

Overall, the results show the multimodal CFP + OCT model had improved performance relative to the unimodal CFP model and comparable performance to the unimodal OCT model and these relationships were held across patient demographics (race, age) and disease severity. There are several potential causes for these results. First, there was a difference in the number of available CFP and OCT images used for training and testing (14 510 CFPs vs. 32 640 OCTs). It is possible the smaller number of CFPs for training limited performance. However, our recent work specifically investigates the number of images needed to maximize RETFound glaucoma detection performance from CFPs using the DIGS/ADAGES data set.^34^ We found that performance gains from adding additional training/fine-tuning images dropped off quickly after a few hundred images. In fact, using a RETFound model fine-tuned on a smaller number of CFPs than considered here (2000 vs. 14 510), we had already achieved nearly identical performance to the unimodal CFP model here (AUC [95% confidence interval]: 0.86 [0.81, 0.89] vs. 0.86 [0.83, 0.88]). While the training/testing images were different across these studies and direct comparison of the models on the same test set cannot be made, it does suggest that performance gains from adding additional images would be small.

The visualization techniques used to highlight image regions that drove model predictions also suggest some relevant differences between the CFP and OCT imaging. The heatmaps were generated for the multimodal CFP + OCT model and highlighted the regions in each image type that impacted predictions. For OCT images, the model consistently focused on anatomical regions known to be relevant to glaucoma, especially the retinal nerve fiber layer as well of some regions near the retinal pigmented epithelium and choroid. For CFPs, heatmaps for the CFPs often highlighted the edges or outer regions of the images rather than focusing on the ONH, particularly in cases where the model misclassified the glaucoma status. This suggests that there may be image artifacts (e.g., dark areas beyond the imaging field of view) or other issues limiting the contributions of CFPs to the multimodal model. These findings also highlight challenges with visualizations to aid AI explainability—the heatmaps provide clues to model decision-making, but additional testing is needed to investigate these issues.

Several limitations of this study should be noted. As mentioned above, the relatively small CFP dataset may be limiting the performance of the unimodal CFP model compared with the others. Although the current CFP performance is similar to our other recent results,^25^ additional experiments with equally sized fine-tuning training sets are needed. In addition, the specific definition of glaucoma used in this study was based on expert review of individual CFPs and functional measurements or longitudinal changes were not used to identify glaucomatous eyes. Despite the CFP-based ground truth, the unimodal CFP model had lower performance than the unimodal OCT or multimodal models. Because we know that the choice of glaucoma definition can have a large impact on resulting model performance,^10^ future research using alternative glaucoma definitions that incorporate additional VF, OCT, and clinical measurements may help give more insight into the performance of the unimodal CFP, OCT, and multimodal models. Another limitation is the specific approaches used to combine the unimodal RETFound models into a single multimodal approach. In this work, we chose a relatively simple approach that allowed us to generate attention maps to aid model explainability. Each unimodal RETFound model was used as a feature extractor for either CFP or OCT images. The extracted features were then concatenated together and fed into a combined classification model consisting of multiple fully connected layers. We recognize that this is only one of many potential multimodal implementations. Future work will investigate other multimodal fusion strategies that could also incorporate additional data modalities. Finally, additional external data sets are needed to further evaluate these models. For this study, we used training and data collected as part of the DIGS/ADAGES which include data collected from diverse patient populations at geographically separated sites across the United States and models trained on this diverse data set have tended to generalize well to new data.^10^ Our independent test set consisted of a random selection of patients from the 3 sites but not an external independent test. To assess generalizability of the model, our stratified analysis showed that patient age and race did not significantly affect the diagnostic accuracy of our unimodal and multimodal AI models. However, application to unseen, external datasets is critical in evaluating AI models such as those described here.

Future work will build upon the current study by addressing these limitations and expanding the data types incorporated into our multimodal approaches. Incorporating additional ophthalmic imaging, functional testing, and health data in multimodal models may improve diagnostic accuracy compared with a unimodal OCT glaucoma detection model. It may also allow other predictions related to glaucoma management (early detection, prediction of progression) as well as detection of systemic diseases. Foundation AI models such as RETFound provide a platform on which clinically impactful tools can be built and future work is needed to explore their potential.

In conclusion, the multimodal RETFound model did not significantly outperform the unimodal OCT model in glaucoma detection. These results suggest that clinical implementation of a unimodal OCT AI model may provide useful clinical decision support for glaucoma management.
