# Supplementary material for: A Novel Multimodal Implementation of a Foundation Artificial Intelligence Model Using Optic Nerve Head Fundus Photographs and OCT Imaging for Glaucoma Detection
Source: Ophthalmol Sci. 2025 Nov 17;6(2):101012. doi: 10.1016/j.xops.2025.101012 (PMC12861149; doi:10.1016/j.xops.2025.101012)
Supplement: Table S1 [file mmc1.docx]

**Supplemental Table 1**

A. Subject, Eye, and Image-level Train+Valid+Test Sample Sizes

| Cohort | Model | Sample Sizes (All Images) | | | Sample Sizes (Most Recent Images Only) | | |
| --- | --- | --- | --- | --- | --- | --- | --- |
|  |  | Subjects | Eyes | Images | Subjects | Eyes | Images |
| Overall | Unimodal CFP | 1098 | 1947 | 14510 | 1098 | 1947 | 1947 |
|  | Unimodal OCT | 1098 | 1924 | 32640 | 1098 | 1924 | 1924 |
|  | Multimodal | 1095 | 1918 | 105176 | 1095 | 1918 | 1918 |
| Black | Unimodal CFP | 427 | 741 | 5680 | 427 | 741 | 741 |
|  | Unimodal OCT | 427 | 732 | 11458 | 427 | 732 | 732 |
|  | Multimodal | 426 | 730 | 39641 | 426 | 730 | 730 |
| White | Unimodal CFP | 597 | 1072 | 8053 | 597 | 1072 | 1072 |
|  | Unimodal OCT | 597 | 1059 | 18755 | 597 | 1059 | 1059 |
|  | Multimodal | 596 | 1056 | 58704 | 596 | 1056 | 1056 |
| Age > 60 | Unimodal CFP | 721 | 1257 | 9849 | 725 | 1268 | 1268 |
|  | Unimodal OCT | 723 | 1244 | 23290 | 728 | 1256 | 1256 |
|  | Multimodal | 720 | 1238 | 75686 | 724 | 1248 | 1248 |
| Age < 60 | Unimodal CFP | 492 | 889 | 4661 | 373 | 679 | 679 |
|  | Unimodal OCT | 453 | 810 | 9350 | 370 | 668 | 668 |
|  | Multimodal | 456 | 817 | 29490 | 371 | 670 | 670 |
| Mod to Adv Glaucoma | Unimodal CFP | 59 | 109 | 645 | 43 | 79 | 79 |
|  | Unimodal OCT | 59 | 103 | 1226 | 42 | 71 | 71 |
|  | Multimodal | 56 | 96 | 4345 | 45 | 76 | 76 |
| Mild Glaucoma | Unimodal CFP | 102 | 178 | 2394 | 80 | 136 | 136 |
|  | Unimodal OCT | 99 | 166 | 3095 | 83 | 135 | 135 |
|  | Multimodal | 94 | 158 | 10341 | 77 | 125 | 125 |

B. Subject, Eye, and Image-level Test Sample Sizes

| Cohort | Model | Sample Sizes (All Images) | | | Sample Sizes (Most Recent Images Only) | | |
| --- | --- | --- | --- | --- | --- | --- | --- |
|  |  | Subjects | Eyes | Images | Subjects | Eyes | Images |
| Overall | Unimodal CFP | 200 | 374 | 4122 | 200 | 374 | 374 |
|  | Unimodal OCT | 200 | 351 | 5797 | 200 | 351 | 351 |
|  | Multimodal | 197 | 345 | 19196 | 197 | 345 | 345 |
| Black | Unimodal CFP | 77 | 142 | 1550 | 77 | 142 | 142 |
|  | Unimodal OCT | 77 | 133 | 2056 | 77 | 133 | 133 |
|  | Multimodal | 76 | 131 | 7690 | 76 | 131 | 131 |
| White | Unimodal CFP | 114 | 216 | 2413 | 114 | 216 | 216 |
|  | Unimodal OCT | 114 | 203 | 3486 | 114 | 203 | 203 |
|  | Multimodal | 113 | 200 | 10620 | 113 | 200 | 200 |
| Age > 60 | Unimodal CFP | 132 | 243 | 2555 | 132 | 243 | 243 |
|  | Unimodal OCT | 132 | 227 | 4072 | 132 | 227 | 227 |
|  | Multimodal | 131 | 224 | 13799 | 131 | 224 | 224 |
| Age < 60 | Unimodal CFP | 113 | 214 | 1567 | 70 | 131 | 131 |
|  | Unimodal OCT | 79 | 145 | 1725 | 69 | 124 | 124 |
|  | Multimodal | 77 | 142 | 5397 | 67 | 121 | 121 |
| Mod to Adv Glaucoma | Unimodal CFP | 59 | 109 | 645 | 43 | 79 | 79 |
|  | Unimodal OCT | 59 | 103 | 1226 | 42 | 71 | 71 |
|  | Multimodal | 56 | 96 | 4345 | 45 | 76 | 76 |
| Mild Glaucoma | Unimodal CFP | 102 | 178 | 2394 | 80 | 136 | 136 |
|  | Unimodal OCT | 99 | 166 | 3095 | 83 | 135 | 135 |
|  | Multimodal | 94 | 158 | 10341 | 77 | 125 | 125 |

C. Non-Glaucomatous Test Sample Sizes

| Cohort | Model | Sample Sizes (All Images) | | | Sample Sizes (Most Recent Images Only) | | |
| --- | --- | --- | --- | --- | --- | --- | --- |
|  |  | Subjects | Eyes | Images | Subjects | Eyes | Images |
| Non-Glaucomatous | Unimodal CFP | 95 | 171 | 1078 | 88 | 159 | 159 |
|  | Unimodal OCT | 83 | 148 | 1476 | 81 | 145 | 145 |
|  | Multimodal | 83 | 147 | 4510 | 81 | 144 | 144 |
